# Assessment of the knowledge, attitudes, and practices (KAP) among UNRWA* health staff in Jordan concerning mental health programme pre-implementation: a cross-sectional study

**DOI:** 10.1186/s13033-020-00386-3

**Published:** 2020-07-29

**Authors:** Yassir Turki, Suha Saleh, Shatha Albaik, Yasmeen Barham, Dorien van de Vrie, Yousef Shahin, Majed Hababeh, Merve Armagan, Akihiro Seita

**Affiliations:** 1 Health Department, United Nations Relief and Works Agency for Palestine Refugees in the Near East (UNRWA), Amman, Jordan; 2 College of Fine Arts, Humanities and Social Sciences, The University of Massachusetts Lowell, Lowell, United States

**Keywords:** Mental health, Primary healthcare, UNRWA, Palestine refugees, Psychosocial support, KAP, MHPSS

## Abstract

**Background:**

Mental health is a major public health priority, especially among refugees. The United Nations Relief and Works Agency for Palestine Refugees (UNRWA) started to integrate mental health and psychosocial support (MHPSS) into its primary healthcare services in Jordan in late 2017. In this study, we aimed to assess of the knowledge, attitudes, and practices (KAP) among UNRWA health staff (HS) in Jordan concerning mental health programme pre-implementation, and their perceived barriers about this MHPSS programme.

**Methods:**

A cross-sectional study was conducted among doctors, dentists, nurses, and midwives who work at 16 of the 25 UNRWA health centres in Jordan. The assessment was made using a validated self-administered questionnaire. Data analysis was performed using SPSS (version 22).

**Results:**

Of the participants, 73% (161 of 220) believed that their knowledge of MHPSS programmes was insufficient, with no significant difference (p = 0·116) between different categories of staff. Furthermore, 88% (194 of 220) said that they needed more training, 67% (147 of 220) reported that the number of mental health cases is increasing, and 50% (110 of 220) that dealing with these cases is difficult. Reflecting on the past 12 months, 31% of staff (69 out of 220) reported meeting between one and ten children, and 45% (100 out of 220) reported meeting between one and ten adults suspected of having mental illnesses. The most suspected condition was depression (84%; 150 of 220), followed by epilepsy (64%; 140 of 220). The main perceived barriers to implementation included the limited availability of MHPSS policies (87%; 192 of 220), MH professionals (86%; 190 of 220), resources (86%; 189 out of 220), and lack of privacy (14%; 31 out of 220).

**Conclusions:**

Most health staff had positive attitudes towards MHPSS programme implementation but felt they lacked the required knowledge. There is a need for training and clear technical guidelines. Perceived barriers to MHPSS programme implementation were consistent with the previous studies and need to be tackled with a structured plan of action.

## Background

### Overview

The World Health Organisation (WHO) defines mental health as “a state of well-being in which every individual realizes his or her own potential, can cope with normal stresses of life, can work productively and fruitfully, and is able to make a contribution to her or his community” [1].

Mental disorders have quickly became one of the leading causes of illness and disability in the world; 450 million people were estimated to be suffering from such disorders in 2001 [2]. In 2015, it was found that out of the 229.2 million Disability Adjusted Life Years (DALYs) in the Eastern Mediterranean Region (EMR), there were 10.7 million DALYs caused by mental disorders, comprising 4.7% of total DALYs, and making mental disorders the ninth leading cause of disease burden in the region [3].

Treatment for mental disorders is available; however, a lack of utilization of the treatment, as well as stigma and discrimination, have made mental disorders’ treatment neglected or underestimated [4]. Zaleska and his colleagues conducted a literature review to investigate the physical health problems refugees experience. Authors concluded that migration is a predictor of tension and we observe worsened mental health among migrants. Thus, we should elaborate this situation from a biopsychosocial perspective as migration is a complex phenomenon and has an impact on migrants’ overall well-being including physical and mental health [5].

Refugees are considered as a more vulnerable group in terms of health when compared to immigrants as they fled from extreme conditions that are likely to be traumatic and they usually experience more severe circumstances in their new home. Especially for Palestine refugees, transgenerational trauma might add more to their burden since it gets carried from generation to generation. It means that, if a traumatic incident led to a psychopathology in a generation of parents, their off-springs are also likely to suffer from similar disturbances and disorders no matter if they were traumatized or not. It might be related to both genetic predisposition and family relations such as identification with parents or dysfunctional family structure [6].

As a result, the integration of mental health services at the level of primary health care is necessary. These services which include diagnosis and treatment provided by trained health staff implementing strategies to prevent mental disorders, leads to (a) the reduction of stigma for patients with mental illness and their families, (b) the protection of human rights, and (c) improved access to health care. This integration also contributes to the decrease of the illness chronicity, the improvement of social integration, and the development of the human resource capacity for mental health [7]. Due to the highly documented burden of mental disorders, the association between many physical problems and mental health problems, and the enormous gap in the treatment of mental health problems, the integration of mental health care into primary health care is anticipated to bring about many benefits. This integration will enhance access to care, promote respect to human rights, be affordable and cost effective, and lead to better health outcomes. Although certain skills and competencies are necessary to effectively deal with the assessment, diagnosis, treatment, support and referral to specialised services of people with mental disorders, it is essential and possible that health staff at the primary care level be properly prepared and supported in their efforts to deal with mental health problems [8].

The integration of mental health programmes into primary health care services is not yet achieved in over 30% of countries, and 25% of countries still have no mental health legislation; therefore, the integration of behavioral health care in primary health care has become an area of focus into health care redesign efforts [9].

Many primary health care facilities all over the world have started to integrate mental health programmes to solve the treatment gap and improve the effectiveness of treatment [4].

### UNRWA and mental health and psychosocial support (MHPSS) services

Worldwide, up to 60% of people attending primary care clinics have a diagnosable mental disorder (WHO and Wonca 2008). Acknowledging that current literature about the psychological health could not be found, the results of one study on Palestinian adult refugees in Jordan showed that 43% of participants exhibited symptoms of major depression associated with poor perception of health and low level of hopefulness about returning to Palestine [10]. Such data indicate that mental illnesses which are very serious should be tackled in order to prevent the situation from becoming worse concerning the health of Palestine refugees in general.

In 2015, UNRWA considered the integration of Mental Health and Psychosocial Support services (MHPSS) into primary health care (PHC) as an Agency-wide priority, utilizing WHO’s mental health Global Action Programme (mhGAP) to deal with patients who are likely to have mental health conditions [11].

At the end of January 2016, Saftawi health centre in North Gaza started what has proven to be a successful pilot to provide MHPSS services to its patients. Based on this success, the agency started, in July 2017, to integrate the tested MHPSS model into its Family Health Team (FHT) approach of service provision, a patient-centred approach to health care in all fields of UNRWA operations. Before the integration of the programme, a plan was set to offer health staff; including medical officers, staff nurses, and midwives, training on psychosocial support and mhGAP to promote their skills to care for patients with mental health issues. Other health staff categories received training on psychosocial support and protection [12].

### Significance of the study

The results of the study are expected to provide the agency with baseline information about the current status of the knowledge, attitudes and practices of health staff regarding mental health at PHC facilities in Jordan before the implementation of an adopted MHPSS programme. Additionally, the results will provide an idea about the perceived barriers to the implementation of this programme. The outcomes of the study will be used as a reference when conducting a post-implementation assessment as a monitoring tool to determine the impact of MHPSS within 12 months after implementation. The outcomes will also provide guidance for the UNRWA health programme management concerning the development of policies and practice guidelines and the content of training that health staff will receive in the future. In addition, this study fills an immense gap in the literature as, to our knowledge, there are no studies that investigate the attitudes, sources of knowledge, practices and barriers while implementing MHPSS program for Palestine refugees in Jordan.

## Methods

### Design and aim of the study

A cross sectional study design was used to explore and to assess the current knowledge, attitudes, and practices (KAP) among health staff at UNRWA health centres in Jordan concerning MHPSS programme pre-implementation, and their perceived barriers to its integration into PHC services.

### Study setting

The study was conducted during August to September 2017. 16 out of a total of 25 UNRWA health centres in Jordan were selected conveniently based on their size and accessibility. Of the sixteen selected health centres, there were 7 large health centres (Jabal Hussein, Main Baqaa, Amman New Camp, Zarqa Camp, Marka, Awajan and Irbid New health centre), 7 medium health centres (Amman Town, Nuzha, South Baqaa, Zarqa Town, Husun, Jerash and Suf), and 2 small sized health centres (Talbieh and Kraimeh).

### Target population

The target population in this study included all medical officers, dentists, nurses (including Senior Staff Nurses, Staff Nurses as well as Practical Nurses) and midwives working at UNRWA health centres in Jordan. The other healthcare workers including; pharmacists, lab technicians, clerks, and cleaners were excluded. They target population comprised a total of 390 personnel. All targeted staff members at the 16 health centres were approached to participate in the study. The actual number of staff who completed the study questionnaire was 220.

### Instrument of the study

For this study, the research team at UNRWA headquarter developed a structured questionnaire based on the literature as well as the World Health Organization Assessment Instrument for Mental Health Systems (WHO-AIMS) version 2.2 [13]. WHO-AIMS (WHO 2005) tool contains a checklist on what needs to be included in a report of the country’s mental health system. However, since UNRWA health services lie within the PHC context, not all the domains described in the checklist were applicable, thus certain domains were selected. The questionnaire was enhanced with more in-depth questions to get a comprehensive overview of the current situation of the health care staff regarding the mental health services and the integration of MHPSS programme.

Content validity was assessed by three experts; research consultant, health communication officer, and the Director of Lisbon Institute for Global Mental Health. Accordingly, content validity index (CVI) was computed by dividing the relevance number over the total number of experts, Therefore, for this study, CVI for equal 1.0, means it has distinguishing relevance to what is intended to measure.

Amir Hasan Health Centre was selected as the site to conduct the pilot study, to test the questionnaire clarity and response time, and to determine if any modifications were needed. The questionnaire was constructed in English, translated into Arabic, and then back-translated into English to ensure the questionnaire was as valid as possible. The validated Arabic version was used as the tool in this study.

The final version of the questionnaire had five main parts;

Part 1: general information

This part was composed of six questions asking the staff about their gender, professional discipline, highest academic degree attained, year of graduation and years of experience inside and outside UNRWA.

Part 2: knowledge about mental health and the status of mental health services at UNRWA PHC centres.

This part asked health staff about their knowledge regarding Mental Health Programme’s policies and plans, the sources for gaining knowledge about mental health and the availability of psychotropic medicines at their health centres. Additionally, the health staff members were asked if they have received training on psychosocial (non-pharmacological) interventions, child and adolescent mental health issues, and the rational use of psychotropic medicines, as well as if they had taken theoretical and practical psychiatry and mental health courses during their college study

Part 3: health staff attitude about mental health programme implementation

This part assessed the health staff’s attitudes toward the implementation of a MHPSS programme. A three-point Likert scale was used for response categories with a rating scale of agree, not sure and do not agree. The original Bloom’s cutoff points were adapted and modified to determine attitude levels among health staff, in which less than 50% was considered as negative (low level) attitude, 50%–70% was considered as neutral (middle level), and above 70% was considered as positive (high level) attitude [14, 15].

Part 4: practices of the health staff regarding mental health services

This part assessed health staff training and practices regarding psychotropic medications prescriptions, patients’ mental health in terms of the number of suspected mental health cases seen, types of cases, and the provision of consultation and referral of cases as appropriate.

Part 5: barriers against mental health programme implementation

This part aimed to assess the degree of agreement of health staff regarding the barriers of implementing an MHPSS programme from their perspective, with an additional open-ended question allowing health staff to suggest other barriers.

### Data collection

The questionnaire in Arabic was distributed to health staff at the selected health centres by UNRWA’s health department research assistants. Staff members were offered enough time, estimated at an average of 15–20 min, to complete the questionnaire before collecting them back.

### Data analysis

Data entry and data analysis were carried out using Statistical Package for the Social Sciences (SPSS) version 22. The codes of the negatively-worded items in the attitude section were reversed before data analysis. Descriptive statistics were used to describe the sample characteristics, in which frequency and percentage were used for categorical variables, while mean and standard deviation were used for numerical variables. Pearson Chi Square test was used to assess the association between general characteristics (gender & professional discipline) and knowledge questions, while Analysis of variance (ANOVA) test was used to assess the association between professional discipline and attitudes scores, and the independent t-test was used to assess the relation between knowledge questions and attitudes items. Significance level was set at ≤ 0.05.

### Ethical considerations

An approval from the senior management at the front office of UNRWA in Jordan was obtained. All participants agreed on a written consent to participate in the study. A statement of confidentiality was included in the cover letter of the questionnaire to assure participants that no disclosure of their identities would be made.

## Results

### General information of the participating health staff

220 health staff from 16 UNRWA health centres in Jordan participated in this study. The majority of the participants were females (74.1%). Of the total, 41.8% of the staff were practical nurses, followed by general practitioners (22.7%). Also, senior/staff nurses (16.8%), midwives (11.4%) and dentists (7.3%) from UNRWA health centers participated in the study. The percentages of the participants with diploma and bachelor’s degrees were 44.1% and 42.3% respectively. Moreover, those who hold a master’s degree were 7.3% of all participants. The mean duration of work experiences after graduation was 21.2 years (± 9.4). 41.4% of the total graduated more than 25 years ago. The mean years of experience outside UNRWA were 7.1 years (± 6.7). The mean years of experience within UNRWA were 13.5 years (± 7.8).

### Knowledge about mental health and the status of mental health services at UNRWA health centres

44.1% of the staff knew about the availability of psychotropic medicines at their health centres, while 34.5% reported that they are not available. 21.4% did not know whether those drugs were available at their health centres or not. Of the total, 73.2% of the participants did not think that they have enough knowledge about Mental Health Programme’s (MHP’s) policies and plans. Most of the staff did not receive training or refresher training regarding relevant mental health topics, while 67.7% of the participants had credit hours (theoretical and practical) dedicated to psychiatry and mental health related subjects during their study at the university/college. On the other hand, our results demonstrated that 85% of participants never received a refresher training on child and adolescent mental health issues. In addition, only 4.5% of the staff received a refresher training on the rational use of psychotropic medicines (Table 1).

**Table 1:** Distribution of the participants based on the questions that assess their knowledge about mental health (n = 220)

Questions	Frequency (%)		
	Yes	No	Don’t know ^a^
1. Are psychotropic medicines available at your health centre?	97 (44.1)	76 (34.5)	47 (21.4)
2. Do you think that you have enough knowledge about Mental Health Programme’s policies and plans?	59 (26.8)	161 (73.2)	
3. Did you receive training about psychosocial (non-pharmacological) interventions?	75 (34.1)	145 (65.9)	
4. Did you receive refresher training on child and adolescent mental health issues?	32 (14.5)	188 (85.5)	
5. Did you receive refresher training on the rational use of psychotropic medicines?	10 (4.5)	210 (95.5)	
6. During your study at the university/college, did you have credit hours (theoretical and practical), which were dedicated to psychiatry and mental health related subjects?	149 (67.7)	71 (32.3)	

^a^ Just for the first question

Our results indicate that there were significant differences (*p *< 0.010) among the participants regarding their knowledge about the availability of psychotropic medicines at their health centres based on gender and on their professional discipline. Males’ knowledge was significantly higher than that of females (68.4% and 35.6% respectively). Additionally, 72% of the general practitioners reported that the psychotropic medicines were available at their health centres which might be an indicative of how well-informed they were when compared to the other professional disciplines as only 4% of them stated that they do not know about the availability of psychotropic drugs. After general practitioners, practical nurses are the most knowledgeable professional discipline since 80.5% of them has information about the availability of those drugs. On the other hand, 50% of the dentists did not know about the availability of those drugs followed by midwives (36%).

We asked the participants whether they were allowed to prescribe psychotropic medicines or not and stratified the results by professional disciplines. Results demonstrated that 64% of general practitioners were allowed to prescribe it. Thus, they were more knowledgeable and well-informed about the psychotropic medicines. However, none of the dentists were allowed to prescribe it and this helps us understand why half of the dentists were not knowledgeable about such medicines. More than 90% of each professional discipline other than general practitioners responded negatively about their ability to prescribe psychotropic medicines.

In the survey, we also asked participants whether they think they have enough knowledge about Mental Health Programme’s policies and plans or not. The majority of the staff members from all the selected disciplines stated that they did not have enough knowledge about MHP’s policies and plans; there was no significant difference (*p *= 0.116) among disciplines regarding this question. However, general practitioners were the most knowledgeable in this particular issue too. According to the results of our present study, 40% of general practitioners answered the question as “yes” whereas it was only 16% for midwives.

Furthermore, we asked about their sources of knowledge regarding mental health. Our results indicated that training courses were the most common source (17.7%) followed equally by university/college courses and dealing with frequent cases (8.6% for each). These were followed by reading books (7.7%), social media (3.6%) and TV (3.2%).

### Health staff attitudes towards the implementation of the MHPSS programme

The participants answered six questions to assess their attitudes towards mental health services. Table 2 shows that a high proportion of the staff agreed with most of the statements. The mean of the overall attitudes’ score was 13.2 (± 1.9).

**Table 2:** Distribution of the participants based on their responses on items that assess their attitudes toward the implementation of the MHPSS programme (n = 220)

Statements	Frequency (%)		
	Agree	Not sure	Don’t agree
1. The number of patients I see with signs and symptoms of mental health problems is increasing	147 (66.8)	62 (28.2)	11 (5)
2. I consider that dealing with patients who have mental health problems is an extra burden on me	122 (55.5)	39 (17.7)	59 (26.8)
3. I think that dealing with patients who have mental health problems is difficult	129 (58.6)	51 (23.2)	40 (18.2)
4. Psychotropic medicines usually result in the improvement of the condition of patients with mental health problems	115 (52.3)	89 (40.5)	16 (7.3)
5. I feel competent to offer counseling to mentally ill patients	65 (29.5)	93 (42.3)	62 (28.2)
6. I feel that I need more training in mental health	194 (88.2)	15 (6.8)	11 (5)

There were no significant differences in the attitudes scores among staff according to their professional discipline. Expected minimum score was 6 and maximum score was 18. Highest score was the general practitioners’ with 13.6 mean and the lowest score belonged to dentists, midwives and senior/staff nurse with a mean of 12.9 for each category.

More than half of the participating staff (61.8%) had a positive attitude toward the implementation of the MHPSS programme, while 37.7% had a neutral attitude, and only 0.5% had a negative attitude towards its implementation.

### Association between knowledge and attitudes of the participating staff regarding mental health

Table 3 shows significant differences between knowledge questions and attitudes’ scores, where the mean attitude scores were significantly higher (*p* < 0.01) in all questions, except for the last question (*p *= 0.046), among the participants whose answers were “yes” regarding knowledge questions.

**Table 3:** Association between knowledge and attitudes regarding mental health programmes' implementation among participating health staff (n = 220)

Questions	Attitudes’ scores mean (±SD) ^a^		*p* value ^b^
	Yes	No	
1. Do you think that you have enough knowledge about Mental Health Programme’s policies and plans?	14.0 (±1.7)	12.9 (±2.0)	< 0.001
2. Did you receive training about psychosocial (non-pharmacological) interventions?	13.7 (± 1.8)	13.0 (± 2.0)	0.005
3. Did you receive refresher training on child and adolescent mental health issues?	14.1 (± 1.6)	13.1 (± 2.0)	0.002
4. Did you receive refresher training on the rational use of psychotropic medicines?	14.8 (± 1.5)	13.2 (± 2.0)	0.006
5. During your study at the university/college, how many credit hours (theoretical and practical) were dedicated to psychiatry and mental health related subjects?	13.4 (± 1.9)	12.9 (± 1.9)	0.046

^a^ Standard deviation

^b^ Independent t-test

### Practices of the participating health staff regarding mental health services

Our study results demonstrated that most of the participating health staff (77.7%) were not allowed to prescribe psychotropic medicines to patients; of them, 26% were general practitioners. More than a half of the staff (56.8%) did not have practical training/internship/residency at the psychiatry ward during/after their study at the university/college. The majority of health staff (81.4%) did not refer patients to mental health professionals and 94.1% stated they did not have a regular contact with mental health services providers outside UNRWA. Of the total, 56.4% reported that they usually counsel patients with mental health problems about their condition.

Besides, we investigated the mental health disorders that the participating staff suspected. Depression was the most reported condition (84.1%) followed by epilepsy (63.6%) and anxiety disorders (53.2%). Even though they are just suspicions rather than professional diagnoses given by a specialist, it signalizes that stress is likely to be one of the main causes of mental health problems Palestine refugees face.

Our results also show that 9.5% of the health staff had regular contact with psychiatrists outside UNRWA, while 6.4% contacted Ministry of Health (MOH) mental health services providers. The purposes of those contacts were either for referrals (8.6%) or for case discussions (4.5%).

### Barriers against mental health programme implementation

More than half of the participants agreed with all the suggested barriers (Table 4) except for the sixth statement, (“Staff stigmatizing patients with mental health illnesses”), with which 43.6% expressed disagreement. The current study results demonstrate additional barriers expressing the participating staffs’ perspectives, with 33.6% of them considering that the unavailability of well trained staff as one of the barriers that hinders the implementation of MHPSS, 22.7% believe that the absence of training courses regarding communication between patients and doctors to be a barrier as well. In addition, lack of privacy (14.1%) and not having mental health clinics (9.5%) were perceived as barriers against the implementation of MHPSS programme by participants.

**Table 4:** Distribution of the participants’ responses based on their agreement level with statements given about suggested barriers that hinder the implementation of the MHPSS programme (n = 220)

Statements	Frequency (%)		
	Agree	Not sure	Don’t agree
1. Limited availability of psychotropic medications	162 (73.6)	43 (19.5)	15 (6.8)
2. Limited availability of mental health professionals	190 (86.4)	27 (12.3)	3 (1.4)
3. Limited availability of mental health policies/plans/technical instructions (guidelines)	192 (87.3)	23 (10.5)	5 (2.3)
4. Limited resources available (time, cost of treatment, lack of insurance for our patients)	188 (85.5)	29 (13.2)	3 (1.4)
5. Negative cultural perspective about mental health (stigma)	164 (74.5)	33 (15)	23 (10.5)
6. Staff stigmatizing patients with mental health illnesses	61 (27.7)	63 (28.6)	96 (43.6)
7. Patients with mental health illnesses and their care-givers hesitant to attend the health centre for care	129 (58.6)	52 (23.6)	39 (17.7)
8. Insufficient coordination with specialist mental health care in the provision of care to people with severe mental disorders	162 (73.6)	44 (20)	14 (6.4)

## Discussion

This cross-sectional study aimed to explore the level of knowledge, attitudes, and practices of mental health services among health staff at UNRWA PHC centres in Jordan prior to the implementation of the MHPSS programme. This study showed that the majority of the participants express positive attitudes toward the implementation of MHPSS, yet lack sufficient knowledge in regards to mental health programmes, and perceived barriers that highlight their need for MHPSS training.

### Knowledge about mental health and the status of mental health services at the health centres

Health staff at UNRWA health centres in Jordan reported not having sufficient knowledge about mental health programmes, policies, and plans. Other studies also showed that health staff at PHC centres had a low level of knowledge about mental health issues [19, 20]. This finding may be due to the fact that most health staff in this study did not receive training about the different aspects of mental health, including both pharmacological and nonpharmacological treatments. Although more than half of staff reported having undergraduate mental health training (67.7%), this training was reportedly not sufficient enough to affect their confidence about their mental health knowledge. This finding is supported by the WHO-AIMS report for Jordan, where it sated that only 2% of the undergraduate training curriculum of medical doctors and 4% of the training of nurses is related to mental health [16]. Moreover, 17.7% of the staff reported that training courses were their main source of information about mental health disorders, followed by universities/colleges courses (8.6%) and contact with frequent mental health-related cases (8.6%). On the contrary, health care providers in Cambodia declared that they had received most of their knowledge about mental illnesses from public media (61.4%), and some gained their knowledge from university/college studies [15]. These are consistent with the findings of a study conducted with PHC providers at public owned health facilities in Cameroon, in which less than half of the participants had undergraduate training and 0.9% of them had further training after graduation; however, only 1.8% were found to be aware about the standards of the depression diagnostic tool [18].

It was found that male staff were more aware about the availability of psychotropic medicines than female staff with a significant difference (*p *< 0.001); and this might be due to the fact that the number of male general practitioners (GPs) is more than female GPs (41 males vs. 9, females). However, 24% of the general practitioners reported that there was no medicines available at their health centres; in addition, 50% of dentists did not even know whether the drugs were available or not despite the fact that the psychotropic drugs are afforded by the agency. The main reason for this disparity among different staff concerning their knowledge level about the availability of psychotropic medicines could be related to their role in prescribing them. In Jordan, the nurses and other primary health care workers (excluding doctors) are not permitted to prescribe psychotropic medications under any circumstance [16]. Such perception needs comprehensive training about mental health services in terms of diagnosis, and treatment to ensure that all staff become aware about MHPSS programme and the availability of psychotropic medicines at UNRWA health centres. According to Mulango et al. few primary health care providers were aware that psychotropic medicines were available at primary health care pharmacies [18].

### Health staff attitude towards the implementation of MHPSS programme

The current study illustrated that health staff at Jordan UNRWA health centres had an overall positive attitude toward the implementing the MHPSS programme as a need to improve patients’ mental health, which is consistent with previous studies [17, 19].

67% of the participating health staff noticed an increasing number of patients suspected to have mental health problems. More than 40% of the health staff in PHC facilities in Cameroon reported the same observation [18].

Furthermore, approximately 56.0% of the staff agreed that dealing with patients with mental health problems is an extra burden on them, and 58.6% declared that dealing with patients with mental illnesses is difficult. These results are consistent with a previous study, in which 74.3% of health care providers in Cameroon agreed that treating patients with depression weighs heavily on them [18].

42.0% of the health staff in this study were unsure about their competency in offering proper counseling to mental health patients, and 88.2% agreed that more training in mental health programmes is necessary to provide effective mental health care. A similar finding was obtained in a previous study where the majority the staff (91%) considered mental health training to be extremely important [19]. Moreover, it is recommended in WHO-AIMS (Jordan report) to implement mhGAP training for primary care workers [16]. Moreover, in the current study, staff members supporting the integration of MHPSS were significantly associated with those who stated that they had sufficient knowledge and previous mental health training. This finding was the same among primary health workers in south Ethiopia [20]. Overall, mental health training is considered to have a significant and positive impact on the levels of knowledge and attitudes of health staff, thus further ensuring successful implementation of mental health treatment [4].

### Practices of the participating health staff regarding mental health services

Mental illnesses like depression, alcohol and substance use disorders, and psychoses with neurological disorders like epilepsy are the most common illnesses worldwide [21]. This trend closely follows the findings in the current study, in which most suspected cases were perceived as depression (84.1%), epilepsy (63.6%) or anxiety disorders (53.2%). Similarly, depression and anxiety were named as the most prevalent mental illnesses among Palestine refugees in a recent study conducted in South Baqaa health centre [22].

### Barriers against mental health programme implementation

This study assessed the staff’s perceived barriers for implementing the MHPSS programme, with most of the staff agreeing that limited availability of psychotropic medications and mental health professionals (73.6% and 86.4% respectively) were barriers against the implementation of the mental health programme. Furthermore, 85.5% believed that there are limited resources for treating mental health illnesses, including time and cost, which is the same barrier perceived by health workers in Cameroon [18]. According to McKell et al. [22] all participants declared that a lack of resources and financial deficits are key barriers to providing effective mental health care to refugees, and due to this deficit, health centres sometimes could not afford psychotropic drugs for patients.

Furthermore, consistent with the results of previous studies, the negative culture of stigmatization and discrimination against individuals with mental illness was also recognized in this study as barriers for the implementation of the MHPSS programme [18, 22].

Approximately 44.0% of participants did not agree that the staff of UNRWA health centres stigmatize patients with mental illnesses. However, in another study [17], two-thirds of the staff (66.7%) considered mentally ill patients unpredictable and dangerous. Therefore, in comparison, UNRWA staff seems to have a more positive attitude toward the integration of the mental health programme into PHC services.

Limitations of this study include the study design, as a cross-sectional study is more susceptible to recall bias and does not show the causal relationship between the variables. Additionally, the study was only conducted in one of the five fields of UNRWA operations (Jordan).

## Conclusions

This descriptive study assessed health staff knowledge, attitudes, practices, and barriers against the implementation of a MHPSS programme at UNRWA PHC health centres in Jordan.

Most of the staff are in need of training and better knowledge about mental health and MHPSS programme’s policies and plans. Health staff concluded that the most common suspected mental illnesses encountered in the health centres include depression, epilepsy, and anxiety disorder. Most of the staff also reported noticing an increase in the number of patients with mental health problems, meaning the staff members are potentially capable of roughly detecting such cases and support the need for such programmes. Over half of the staff members believe that patients with mental health problems are added burdens for them and that treating patients with such problems is difficult. This finding may be a factor that acts against the acceptance of the implementation of the MHPSS programme. Nevertheless, approximately two-thirds of the health staff expressed a high level of attitude toward implementing a MHPSS programme.

While the majority of the health staff members expressed a high level of positive attitude, they also expressed concerns of multiple barriers against the implementation of MHPSS, including the limited availability of resources (mental health professionals, time, and psychotropic medications). Additionally, stigmatization and discrimination against Palestine refugee patients facing mental illnesses remain unsolved issues that prevent patients from seeking mental health support and prevent the health staff from offering greater support for a MHPSS programme implementation.

There are some recommendations that UNRWA health system needs to take care of in order to implement MHPSS better. Firstly, provision of training on mhGAP & Prevention/Promotion, Assessment, Intervention and Referral (PAIR) models, as well as the continuous education for all concerned staff (refresher courses) on MPHSS is necessary. Secondly, there needs to be a distribution of the finalized Technical Instructions/Guidelines (TIs/TGs) for MHPSS to all concerned staff, and UNRWA should provide them with proper training on them. Thirdly, UNRWA health system should set a plan for continuous monitoring and evaluation of the implementation of MHPSS TIs/TGs.

Also, it is significant to establish learning communities among staff/FHTs on MHPSS at all health centres as it is important to improve the overall attitude of staff concerning mental health programme. For instance, UNRWA might empower staff on MHPSS services provision, and convince them that providing psychosocial support and dealing with mental health conditions will not add extra burden on them, neither it is hard. It is recommended to focus on improvement of the overall cultural perspective of staff and the community about mental health conditions via suitable means. There should be an act on destigmatizing mental health patients among health staff and community. Thus, families/care-givers should be encouraged to bring a family member to the health centre if he/she has a mental health condition.

## Data Availability

The datasets used and/or analysed during the current study are available from the corresponding author on reasonable request.

## Abbreviations

ANOVA: Analysis of variance
DALYs: Disability Adjusted Life Years
EMR: Eastern Mediterranean Region
FHT: Family Health Team
GP: General Practioner
HC: Health centre
HS: Health staff
KAP: Knowledge, attitudes and practices
mhGAP: Mental health Global Action Programme
MHPSS: Mental Health and Psychosocial Support
PAIR model: Prevention/Promotion, Assessment, Intervention and Referral model
PHC: Primary Health Care
SPSS: Statistical Package for the Social Sciences
TGs: Technical Guidelines
TIs: Technical Instructions
UNRWA: United Nations Relief and Works Agency for Palestine refugees in the Near East
WHO: World Health Organisation
WHO-AIMS: World Health Organization-Assessment Instrument for Mental Health Systems

## References

[1] World Health Organization. Promoting mental health: concepts, emerging evidence, practice: Summary report. http://www.who.int/mental_health/evidence/en/promoting_mhh.pdf.

[2] World Health Organization (2001). The World Health Report 2001: mental health: new understanding, new hope. http://www.who.int/whr/2001/en/whr01_en.pdf?ua=1.

[3] Mokdad AH, Charara R, El Bcheraoui C, Khalil I, Moradi-Lakeh M, Afshin A, Kassebaum NJ, Collison M, Krohn KJ, Chew A, Daoud F (2018). The burden of mental disorders in the Eastern Mediterranean region, 1990–2015: findings from the global burden of disease 2015 study. Int J Public Health..

[4] Ayano G, Assefa D, Haile K, Chaka A, Haile K, Solomon M, Yohannis K, Adane AA, Jemal K (2017). Mental health training for primary health care workers and implication for success of integration of mental health into primary care: evaluation of effect on knowledge, attitude and practices (KAP). Int J Mental Health Syst..

[5] Záleská V, Brabcová I, Vacková J (2014). Migration and its impact on mental and physical health: social support and its main functions. Kontakt..

[6] Harkness LL. Transgenerational transmission of war-related trauma. International handbook of traumatic stress syndromes 1993. Springer, Boston. p. 635–643.

[7] World Health Organization (2007). Integrating mental health services into primary health care. http://www.who.int/mental_health/policy/services/3_MHintoPHC_Infosheet.pdf/.

[8] World Health Organization (2008). Integrating mental health into primary care: a global perspective. http://www.who.int/mental_health/resources/mentalhealth_PHC_2008.pdf.

[9] Dickinson WP (2015). Strategies to support the integration of behavioral health and primary care: what have we learned thus far?. J Am Board Family Med..

[10] Alduraidi H, Waters CM (2018). Depression, perceived health, and right-of-return hopefulness of palestinian refugees. J Nurs Scholarsh.

[11] United Nations Relief and Works agency for Palestine Refugees in the Near East (2015). Health Department Annual Report. https://www.unrwa.org/sites/default/files/content/resources/health_department_annual_report_2015.pdf.

[12] United Nations Relief and Works agency for Palestine Refugees in the Near East (2017). Health Department Annual Report. https://www.unrwa.org/sites/default/files/content/resources/health_programme_annual_report_2017.pdf.

[13] World Health Organization (2005). World Health Organization Assessment Instrument for Mental Health Systems. http://www.who.int/mental_health/evidence/AIMS_WHO_2_2.pdf.

[14] Bloom BS, Engelhart MD, Furst EJ, Hill WH, Krathwohl DR (1956). Taxonomy of educational objetives: the classification of educational goals: handbook I: cognitive domain.

[15] Koo HC, Poh BK, Ruzita AT (2015). Assessment of knowledge, attitude and practice towards whole grains among chil-dren aged 10 and 11 years in Kuala Lumpur. Int J Food Sci Nutr Diet..

[16] World Health Organization. (2011). WHOAIMS report of mental health system in Jordan.

[17] Alfredsson M, Sebastian SM, Jeghannathan B (2017). Attitudes towards mental health and the integration of mental health services into primary health care: a cross-sectional survey among health-care workers in Lvea Em District, Cambodia. Global Health Action..

[18] Mulango ID, Atashili J, Gaynes BN, Njim T (2018). Knowledge, attitudes and practices regarding depression among primary health care providers in Fako division, Cameroon. BMC Psychiatry..

[19] Mwape L, Sikwese A, Kapungwe A, Mwanza J, Flisher A, Lund C, Cooper S (2010). Integrating mental health into primary health care in Zambia: a care provider’s perspective. Int J Mental Health Syst..

[20] Abera M, Tesfaye M, Belachew T, Hanlon C (2014). Perceived challenges and opportunities arising from integration of mental health into primary care: a cross-sectional survey of primary health care workers in south-west Ethiopia. BMC Health Services Res..

[21] Murray CJ, Vos T, Lozano R, Naghavi M, Flaxman AD, Michaud C, Ezzati M, Shibuya K, Salomon JA, Abdalla S, Aboyans V (2012). Disability-adjusted life years (DALYs) for 291 diseases and injuries in 21 regions, 1990–2010: a systematic analysis for the Global Burden of Disease Study 2010. Lancet..

[22] McKell C, Hankir A, Abu-Zayed I, Al-Issa R, Awad A (2017). Barriers to accessing and consuming mental health services for palestinians with psychological problems residing in refugee camps in Jordan. Psychiatria Danubina..

